# Changes in the Frequency of Moderate-to-Vigorous Physical Activity and Subsequent Risk of All-Cause and Cardiovascular Disease Mortality

**DOI:** 10.3390/ijerph19010504

**Published:** 2022-01-03

**Authors:** Young Choi, Jae Woo Choi

**Affiliations:** 1 Department of Health Care Management, Catholic University of Pusan, Busan 46265, Korea; choiyoung223@gmail.com; 2 Community Care Research Center, Health Insurance Research Institute, National Health Insurance Service, Wonju 26464, Korea

**Keywords:** sedentary behavior, exercise, moderate-to-vigorous physical activity, heart disease risk factors, cardiovascular diseases, mortality

## Abstract

We investigated the association of changes in the frequency of moderate-to-vigorous physical activity (MVPA) and the risks of all-cause and cardiovascular disease (CVD) mortality. This study used the nationally representative National Health Insurance Service-National Sample Cohort database. We included 286,402 individuals aged ≥20 years and estimated changes in the frequency of MVPA over a two-year period. Hazard ratios (HRs) and 95% confidence intervals (CIs) were calculated using Cox proportional hazard regression models. The HRs (95% CIs) for the risk of all-cause and CVD mortality for an increased frequency of MVPA from physical inactivity compared with continual physical inactivity were 0.82 and 0.68 (0.73–0.92 and 0.51–0.91) for 1–2, 0.72 and 0.48 (0.62–0.84 and 0.31–0.74) for 3–4, and 0.73 and 0.70 (0.63–0.85 and 0.50–0.98) for ≥5 sessions of MVPA/week. The HRs (95% CIs) for the risk of all-cause and CVD mortality were 1.28 and 1.58 (1.07–1.53 and 1.01–2.46), 1.25 and 2.17 (1.01–1.57 and 1.14–4.12), and 1.43 and 1.44 (1.15–1.77 and 0.84–2.47) for changes from 1–2, 3–4, and ≥5 sessions of MVPA/week to physical inactivity, respectively. This study showed the beneficial effect of increasing physical activity, particularly for those who were physically inactive at baseline, as well as the increased risk of all-cause and CVD mortality after adapting a physically inactive lifestyle regardless of their baseline physical activity status.

## 1. Introduction

Physical inactivity is a global pandemic that contributes to a substantial disease and economic burden worldwide. Moreover, insufficient physical activity is one of the leading global risk factors for death [1,2]. A recent pooled analysis of 358 population-based surveys with 1.9 million participants reported that the global age-standardized prevalence of insufficient physical activity was 27.5% (95% uncertainty interval 25.0–32.2%) in 2016 and that levels of insufficient activity remained stable between 2001 (28.5%, 23.9–33.9%) and 2016. A recent Korean analysis revealed that the age-standardized prevalence of physical inactivity has increased from 41.7% in 2014 to 52.4% in 2018 [3]. To promote individual physical activity, there is a need for continued research on the benefits (hazards) of physical activity (inactivity) in health.

The preventative association of moderate-to-vigorous physical activity (MVPA) with all-cause and cardiovascular disease (CVD) mortality has been well established [4,5,6,7]. However, the largest population-based studies documenting this association have examined only one baseline measurement of MVPA and have related this to the subsequent risk of all-cause and CVD mortality. Many people change their levels of physical activity throughout life, and these changes may affect the risk of mortality [8]. Schnohr et al. (2003) examined the association between changes in leisure-time physical activity with the risk of death among 7023 adults [9]. Byberg et al. (2009) examined how changes in the level of leisure time physical activity influence mortality, but they restricted their study to 2205 men aged 50 [10]. Petersen et al. (2012) explored the relationship between changes in physical activity during leisure time and the risk of all-cause mortality in 10,443 adults [8]. Schnohr et al. (2017) investigated the impact of persistence and non-persistence in leisure time physical activity on all-cause mortality [11]. Kieffer et al. (2019) observed the association between temporal changes of physical activity and mortality among 24,890 adults [12]. However, those previous studies could not consider the effects of changes in the frequency of MVPA on mortality due to their relatively small samples; thus, little is known about the health benefits and disadvantages of changes in the frequency of MVPA.

This study examines the association of the frequency of MVPA at baseline and changes in the frequency of MVPA with subsequent all-cause and CVD mortality among Korean adults using a large nationwide cohort data.

## 2. Methods

### 2.1. Data and Study Sample

This study used the nationally representative National Health Insurance Service-National Sample Cohort (NHIS–NSC) database. The sample was randomly stratified and selected based on age, sex, income, and residential region at 2006. Briefly, a nationally representative sample of one million individuals (about 2% of the whole population) was constructed in 2006 from all NHIS enrollees and including all ages. The NHIS–NSC possesses information on the socio-demographic characteristics, health examinations, medical records, and deaths between 2002 and 2015 of the samples. A detailed cohort profile of the NHIS–NSC was published previously [13].

**Figure 1:**
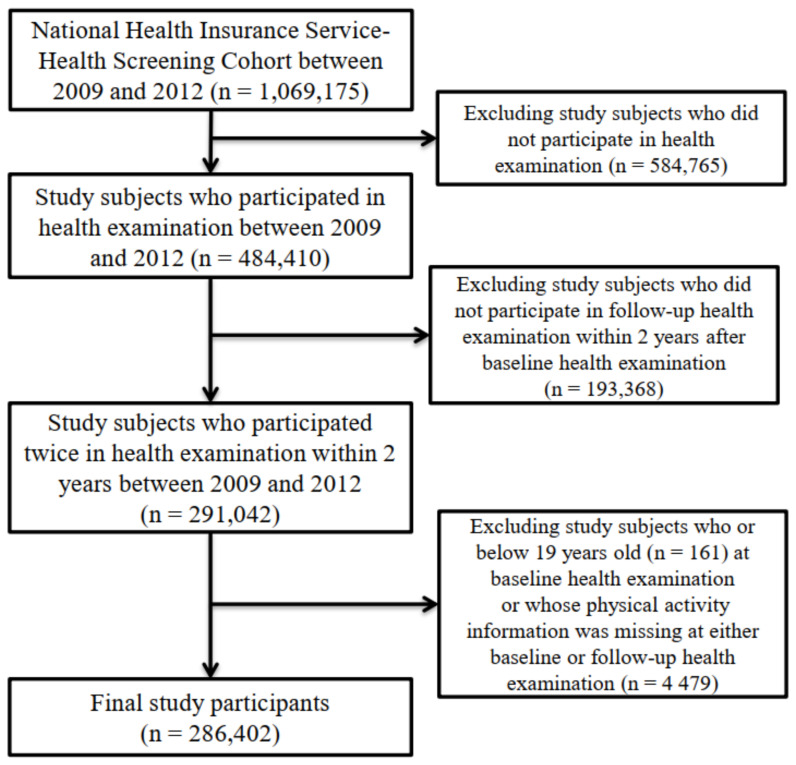
Flow chart of the study participants.

Of the 1,069,175 study subjects included in the NHIS–NSC from 2009 to 2012, we eliminated study participants who did not undergo health screening (n = 584,765) because information on MVPA, which was the variable of interest in this study, was included in the health examination data. We excluded study participants who did not undergo a follow-up health examination (2010–2012) within two years following baseline health screening (2009–2011) (n = 193,368) to capture changes in MVPA. We eliminated those who were younger than 19 years old (n = 161) at the baseline health screening or whose physical activity information was missing at either baseline or during the follow-up health screening (n = 4479). Finally, 286,402 participants were included in this study (Figure 1).

### 2.2. Measurements for All-Cause and CVD Mortality

The dependent variables in this study were all-cause and CVD mortality. The NHIS–NSC data were linked to the death registration database of Statistics Korea, which includes the causes and dates of mortality. The various causes of death were coded according to the International Classification of Disease 10th Revision (ICD-10). In the cause of death records, the ICD-10 codes I00–I99 were defined as CVD mortality.

### 2.3. Measurements for MVPA

The NHIS–NSC data included information about various self-reported questionnaires such as physical activity and other lifestyle behaviors at each national health examination among the participants. Based on the information in the NHIS–NSC, this study utilized records on the number of moderate (≥30 min per day; e.g., brisk walking, tennis doubles, cycling at a medium pace) or vigorous (≥20 min per day; e.g., running, aerobic, fast cycling) physical activity sessions per week at the two consecutive biennial health examinations (2009–2012) to capture changes in the frequency of MVPA among the study subjects. This study created the following categories for the frequency of MVPA: (1) physical inactivity, (2) one to two MVPA sessions per week, (3) three to four MVPA sessions per week, and (4) more than five MVPA sessions per week. We classified changes in the categories from MVPA at the baseline health screening to MVPA at the follow-up health screening. For example, study subjects who were originally physically inactive at the baseline health screening were classified as follows according to their MVPA at the follow-up health screening: (1) remained physically inactive, (2) physical inactivity increased to one to two sessions of MVPA per week, (3) physical inactivity increased to three to four sessions of MVPA per week, and (4) physical inactivity increased to more than five sessions of MVPA per week. The reliability and validity of the questionnaire for physical activity in the NHIS–NSC were explained in a previous study [14].

### 2.4. Measurement for Potential Confounding Factors

Potentially confounding factors consisted of sex, age, body mass index (BMI), systolic blood pressure (SBP), diastolic blood pressure (DBP), serum glucose, total cholesterol, alcohol consumption, cigarette smoking status, household income, location of residence, disability, and comorbidities. Physical measurements including height and weight, blood pressure, and blood tests are performed according to the national health examination guidelines. Height and weight were measured by a body measuring instrument, and BMI was calculated as the weight in kilograms divided by the squared height in meters and divided into groups as follows: <18.5 kg/m^2^ (underweight), 18.5–22.9 kg/m^2^ (normal weight), 23.0–24.9 kg/m^2^ (overweight), 25.0–29.9 kg/m^2^ (class I obese), and ≥30 kg/m^2^ (class II obese) based on the World Health Organization’s recommendations for Asian populations [15]. Blood pressure is measured using an upper arm pressure sphygmomanometer after the examinee rests for at least 5 min. If the systolic blood pressure is 120 mmHg or higher or the diastolic pressure is 80 mmHg or higher, repeat the measurement after an interval of 2 min or more. Serum glucose and total cholesterol levels are measured by enzymatic method after confirming the examinee’s fasting state and then drawing blood with a disposable syringe or vacuum test tube including a disposable needle. Individuals who consumed ≥30 g/day of alcohol were defined as heavy drinkers [16], those who drank alcohol but not heavily were defined as non-heavy drinkers, and those who did not drink alcohol were defined as non-drinkers. Cigarette smoking status was classified as current smoking, ex-smoking, or never smoking. Household income was categorized as follows: (1) low (<40th percentile), (2) middle (41st–80th percentile), or (3) high (81st–100th percentile). Location of residence was defined as metropolitan (capital), urban (local government with >1 million individuals), or rural (other). All confounding factors excluding comorbidities were measured at the date of the follow-up health screening. Diabetes mellitus (ICD-10 codes: E10–E14), hypertension (ICD-10 codes: I10–I15), dyslipidemia (ICD-10 code: E78), ischemic heart disease (ICD-10 codes: I20–I25), stroke (ICD-10 codes: I60–I63), and cancer (ICD-10 codes: C00–C99) were selected as comorbidities and were measured by screening information from all medical records prior to the follow-up health examination.

### 2.5. Statistical Analyses

The characteristics of the study participants at the baseline health screening and follow-up health screening were measured using n (%) for categorical variables and means (standard deviations) for continuous variables. For each study subject, the length of follow-up was measured in days, and all study participants were followed from the date of the follow-up health screening until all-cause mortality or CVD mortality, or the end of 2015, whichever occurred first.

Cox proportional hazards regression models were used to estimate the association between changes in the frequency of MVPA and the subsequent risk of all-cause and CVD mortality. The adjusted hazard ratios (HRs) and 95% confidence intervals (CIs) for mortality were calculated after adjusting for the following variables in the Cox proportional hazards regression models: sex, age, BMI, SBP, DBP, serum glucose, total cholesterol, alcohol consumption, cigarette smoking status, household income, location of residence, disability, and comorbidities. First, we assessed the association between the categories (physical inactivity, one to two times per week, three to four times per week, and more than five times per week) regarding the frequency of MVPA at the baseline health examination with all-cause and CVD mortality. We included the covariates measured at the baseline health examination in the Cox regression models. Second, we analyzed the associations between changes in the categories from MVPA at the baseline health screening to MVPA at the follow-up health screening with all-cause and CVD mortality and utilized confounding factors estimated at the follow-up health examination in the Cox regression models. We also performed sensitivity analyses to determine the associations after eliminating cases of mortality during the first year after the follow-up health examination (Supplementary Table S1).

All data extractions and statistical analyses were conducted utilizing SAS v9.4 (SAS Institute Inc., Cary, NC, USA). Proportional hazards assumptions were evaluated statistically and satisfied for all models. This study was approved by the Yonsei University Institutional Review Board (approval number: 7001988-202006-HR-905-01E), and the requirements for informed consent were waived because the NHIS–NSC data were constructed after anonymization by rigorous confidentiality guidelines. All methods were performed in accordance with all applicable institutional and governmental regulations concerning the ethical use of human participants.

## 3. Results

This study identified 4494 and 820 incident cases of all-cause and CVD mortality during a median follow-up period of 6.1 ± 1.1 years from baseline and 4.3 ± 1.5 years from the follow-up health screening, respectively. The median interval between baseline and the follow-up health examination was 1.6 ± 1.0 years. Table 1 presents the general characteristics of the study participants who received two consecutive biennial national health screenings in 2009–2012. At the follow-up health examination, the mean age (standard deviation) was 50.4 (14.2) years, and 152,572 (53.3%) of the subjects were men. Approximately half of the individuals at the baseline and follow-up health screening periods responded that they were physically inactive (47.9% at the baseline and 45.8% at the follow-up period, respectively). The proportion of MVPA at the follow-up health examination (54.2%) was higher than that at the baseline health examination (52.1%). The other characteristics of the study subjects who underwent the two consecutive biennial health examinations from 2009 to 2012 are presented in Table 1.

Table 2 indicates the HR and 95% CIs for the risks of all-cause and CVD mortality in relation to the frequency of MVPA at baseline. After adjusting for sex, age, BMI, SBP, DBP, serum glucose, total cholesterol, alcohol consumption, cigarette smoking status, household income, location of residence, disability, and comorbidities, a reduced risk of all-cause mortality was observed for one to two (HR, 0.82; 95% CI, 0.76, 0.89), three to four (HR, 0.87; 95% CI, 0.79, 0.96), and more than five (HR, 0.80; 95% CI, 0.73, 0.88) sessions of MVPA per week compared with physical inactivity. A significantly decreased risk of CVD mortality was observed for three to four sessions of MVPA per week (HR, 0.70; 95% CI, 0.55, 0.90) and more than five sessions of MVPA per week (HR, 0.69; 95% CI, 0.54, 0.88) compared with physical inactivity. There was no significant association for one to two sessions of MVPA per week (HR, 0.88; 95% CI, 0.73, 1.07) with the risk of CVD mortality compared with physical inactivity.

**Figure 2:**
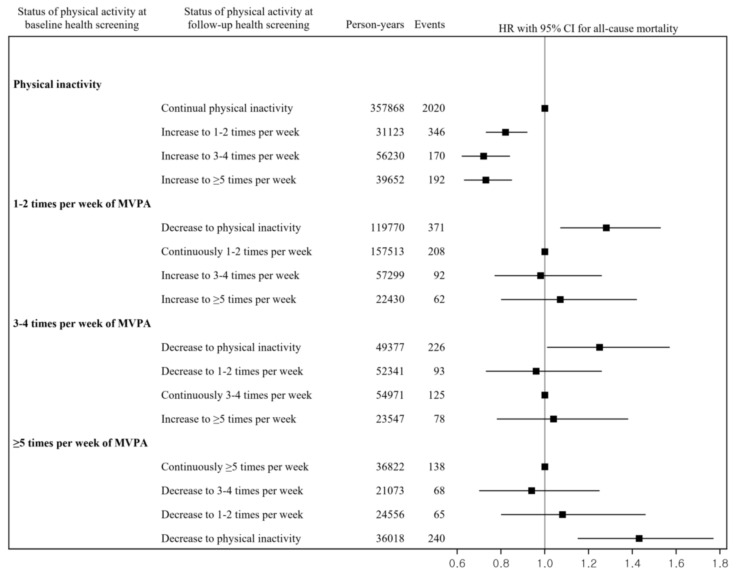
Relationship between changes in the frequency of MVPA and the subsequent risks of all-cause mortality. Note. HR, hazard ratio; CI, confidence interval; MVPA, moderate to vigorous physical activity; CVD, cardiovascular disease. HRs and 95% CIs were estimated after adjusting for sex, age, body mass index, systolic blood pressure, diastolic blood pressure, serum glucose, total cholesterol, alcohol consumption, cigarette smoking status, household income, location of residence, disability, and comorbidities.

**Figure 3:**
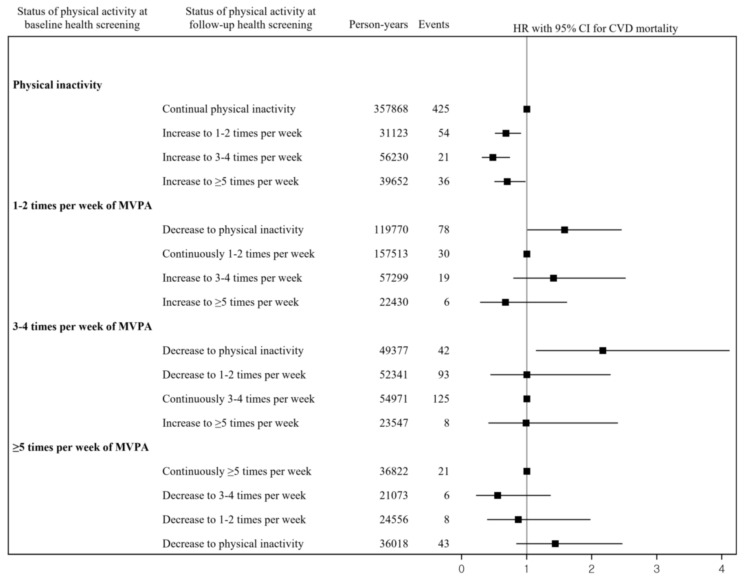
Relationship between changes in the frequency of MVPA and the subsequent risks of CVD mortality. Note. HR, hazard ratio; CI, confidence interval; MVPA, moderate to vigorous physical activity; CVD, cardiovascular disease. HRs and 95% CIs were estimated after adjusting for sex, age, body mass index, systolic blood pressure, diastolic blood pressure, serum glucose, total cholesterol, alcohol consumption, cigarette smoking status, household income, location of residence, disability, and comorbidities.

Figure 2 and Figure 3 show the adjusted HRs and 95% CIs for the associations between changes in the frequency of MVPA with the risks of all-cause and CVD mortality. The HRs (95% CIs) for the risks of all-cause and CVD mortality compared with remaining physically inactive were 0.82 and 0.68 (0.73, 0.92 and 0.51, 0.91) for the increase in MVPA from physical inactivity to one to two sessions per week, 0.72 and 0.48 (0.62, 0.84 and 0.31, 0.74) for the increase in MVPA from physical inactivity to three to four sessions per week, and 0.73 and 0.70 (0.63, 0.85 and 0.50, 0.98) for the increase in MVPA from physical inactivity to more than five sessions per week, respectively. The HRs (95% CIs) for the risks of all-cause and CVD mortality compared with maintaining one to two sessions of MVPA per week were 1.28 and 1.58 (1.07, 1.53 and 1.01, 2.46) for the decrease in MVPA from one to two sessions per week to physical inactivity, 0.98 and 1.41 (0.77, 1.26 and 0.79, 2.52) for the increase in MVPA from one to two sessions per week to three to four sessions per week, and 1.07 and 0.67 (0.80, 1.42 and 0.28, 1.62) for the increase in MVPA from one to two sessions per week to more than five sessions per week.

The HRs (95% CIs) for the risks of all-cause and CVD mortality compared with maintaining three to four sessions of MVPA per week were 1.25 and 2.17 (1.01, 1.57 and 1.14, 4.12) for the decrease in MVPA from three to four sessions per week to physical inactivity, 0.96 and 1.00 (0.73, 1.26 and 0.44, 2.29) for the decrease in MVPA from three to four sessions per week to one to two sessions per week, and 1.04 and 0.99 (0.78, 1.38 and 0.41, 2.40) for the increase in MVPA from three to four sessions per week to more than five sessions per week. The HRs (95% CIs) for the risks of all-cause and CVD mortality compared with maintaining more than five sessions of MVPA per week were 0.94 and 0.55 (0.70, 1.25 and 0.22, 1.37) for the decrease in MVPA from more than five sessions per week to three to four sessions per week, 1.08 and 0.87 (0.80, 1.46 and 0.39, 1.98) for the decrease in MVPA from more than five sessions per week to one to two sessions per week, and 1.43 and 1.44 (1.15, 1.77 and 0.84, 2.47) for the decrease in MVPA from more than five sessions per week to physical inactivity.

## 4. Discussion

This study examined the association between MVPA and risks of all-cause and CVD mortality using a nationwide cohort study. The results showed that participants who were moderately or vigorously physically active at baseline had a reduced risk of all-cause and CVD mortality compared to those who were physically inactive. Moreover, this study investigated changes in the frequency of MVPA associated with all-cause and CVD mortality risks. This study showed that increasing the frequency of MVPA was associated with decreased risks of all-cause and CVD mortality. By contrast, decreases to physical inactivity from 1–2, 3–4, and ≥5 times MVPA per week were associated with an increased risk of all-cause and CVD mortality.

Our findings indicating a lower risk of all-cause mortality in adults who performed MVPA are consistent with previous results. Moore et al. (2012) found that leisure time MVPA was associated with a gain of 1.8–4.5 (95% CI: 1.6–4.7) in life expectancy relative to no leisure time activity [5]. Gebel et al. (2015) revealed that the adjusted HR for all-cause mortality in adults who performed MVPA was 0.46–0.66 (95% CI, 0.43-0.71) compared with those who reported no MVPA [4].

Furthermore, our study found an inverse association between physical activity and CVD mortality, which was consistent with previous studies. Barengo et al. (2004) reported that CVD mortality (9–27%) was lower in adults who were moderately or highly physically active during occupational or leisure time [7]. Zhao et al. (2019) showed that adults who performed leisure time MVPA had a reduced risk of CVD mortality (24–49%) compared with inactive individuals [6]. These inverse associations may be attributed to improved lipid profiles and insulin sensitivity, reduced blood pressure, improved endothelial function, and reduced oxidative stress [17,18,19,20,21,22,23,24,25,26]. The consistency of our findings with previous results suggests that MVPA is associated with reduced deaths from CVD and all-cause mortality.

When assessing changes in MVPA, previous studies primarily focused on the risk of mortality from changes in the intensity of physical activity. Schnohr et al. (2003) reported that adults who consistently engaged in a moderate or high degree of physical activity or who increased their leisure-time physical activity from low to moderate or high had significantly lower risks of death than those reporting low activity at both examinations [9]. Byberg et al. (2009) showed that increased physical activity during middle-age is eventually followed by a reduction in mortality to the same level as that observed among men with a consistently high level of physical activity [10]. Petersen et al. (2012) indicated that men who increased their physical activity had a lower risk of mortality, and both men and women who reduced their physical activity had a higher risk of mortality compared to an unchanged physical activity level [8]. Schnohr et al. (2017) showed that a small or significant decrease in the intensity of leisure time physical activity compared with maintaining a consistent leisure time physical activity level was associated with a higher risk of all-cause and coronary heart disease mortality [11]. Kieffer et al. (2019) revealed that adults who were continuously active enough or those who changed from being inactive to being sufficiently active had lower risks of all-cause and CVD mortality [12]. Previous studies that examined the association between increased frequency of physical activity and a lower risk of mortality were shown with similar results of our study. Our findings highlight that an increased frequency of MVPA is associated with a reduced risk of all-cause and CVD mortality.

This study showed non-linear results between changes in physical activity and mortality. Although there was a significant association with outcomes when people started or stopped being physically active, changes in frequency (decrease or increase) among those who were physically active did not show a significant association with outcomes. These results suggest that a simple change in frequency among people who are continuously physically active may not have an effect on mortality. Further research is needed to determine whether changes in the frequency of physical activity among people who were physically active have an effect on major diseases.

Our study has several limitations. First, since information on frequency and intensity of physical activity was surveyed from self-reported questionnaires, our study on assessing changes in the frequency of MVPA may include possible misclassifications or reporting bias. In addition, questionnaires for physical activity in the NHIS–NSC lacked data on precise performance time or duration of MVPA and information on specific types of MVPA. Second, relatively short follow-up periods may invoke reverse causality, i.e., there is a potential that underlying (diagnosed or undiagnosed) illnesses negatively affect physical activity, so the observed association between physical activity and mortality may be more driven by the causal link between the underlying disease state and subsequent risk of death rather than the physical activity exposure per se [27]. This will typically lead to an overestimation of the true association between physical activity and mortality. Common methods to consider this bias include removing those who experience an event soon after baseline [28]. We performed sensitivity analyses for these associations after eliminating cases of mortality during the first year after the follow-up health examination, and the results were similar to our main findings, although the strengths for the associations were slightly weakened. Further studies on the association between changes in the frequency of MVPA with all-cause and CVD mortality are warranted in data with longer follow-up periods. Third, there were unmeasured or residual confounding factors, as with other studies that have used administrative claims data. Thus, it should be careful in interpreting the results of this study. Finally, this study included Korean adults over 20 years old who participated in two consecutive biennial national health screenings among a representative population. The study subjects might have been more health conscious than those not having a health examination. Nevertheless, the proportions of physical activity, current smoking, and alcohol consumption were comparable with that reported in KNHANES in Korea [29]. Further study is warranted to explore the associations between changes in the frequency of MVPA and mortality in other ethnicities or countries.

In conclusion, the increase in the frequency of MVPA per week was associated with a significant risk reduction of all-cause and CVD mortality among those who were physically inactive at baseline. Our findings also imply that changes from 1–2, 3–4, and ≥5 sessions of MVPA per week to physical inactivity were associated with an increased risk of all-cause and CVD mortality, respectively. These results were shown in association between more frequency of MVPA and decreased risk of premature death from any cause or CVD. To prevent all-cause and CVD mortality, physical activity should be approached as a public health issue. It focuses on the basic principles of public health surveillance, evidence-based and proven strategies. For example, there is a need to support policies to increase green spaces, parks, and walkable communities, working with multi-sectoral sectors such as transport and urban planning [30].

## Figures and Tables

**Table 1:** General characteristics of study participants who received two consecutive biennial national health screenings in 2009–2012.

Variables		Baseline Health Examination (2009–2011)		Follow-Up Health Examination (2010–2012)	
		N	%	N	%
Total		286,402	100.0	286,402	100.0
Physical activity					
	Physically inactive	137,181	47.9	131,286	45.8
	1–2 sessions of MVPA per week	80,598	28.1	82,822	28.9
	3–4 sessions of MVPA per week	41,178	14.4	44,087	15.4
	≥5 sessions of MVPA per week	27,445	9.6	28,207	9.8
Sex					
	Men	152,572	53.3	152,572	53.3
	Women	133,830	46.7	133,830	46.7
Age (years), mean ± SD		48.9	14.0	50.4	14.2
BMI (kg/m^2^)					
	≤18.5	10,045	3.5	9802	3.4
	18.5–23	110,139	38.5	108,920	38.0
	23–25	71,339	24.9	71,832	25.1
	25–30	84,671	29.6	85,226	29.8
	≥30	10,092	3.5	10,571	3.7
SBP (mmHg), mean ± SD		122.4	14.9	122.4	14.8
DBP (mmHg), mean ± SD		76.2	10.0	76.2	9.9
Fasting glucose (mg/dL), mean ± SD		97.3	23.2	97.8	22.9
Total cholesterol (mg/dL), mean ± SD		195.3	40.9	195.0	38.3
Alcohol consumption					
	None	148,456	51.8	151,314	52.8
	Heavy drinking	18,574	6.5	17,663	6.2
	Non-heavy drinking	118,284	41.3	117,293	41.0
Cigarette smoking status					
	Never smoking	174,559	60.9	175,051	61.1
	Ex-smoking	42,294	14.8	45,212	15.8
	Current smoking	68,804	24.0	65,955	23.0
Household income					
	Low	90,640	31.6	89,532	31.3
	Middle	122,187	42.7	121,647	42.5
	High	73,575	25.7	75,223	26.3
Location of residence					
	Metropolitan	53,995	18.9	53,090	18.5
	Urban	75,644	26.4	75,180	26.2
	Rural	156,763	54.7	158,132	55.2
Disability		14,816	5.2	16,461	5.7
Comorbidities					
	Diabetes mellitus	60,883	21.3	71,404	24.9
	Hypertension	78,817	27.5	89,089	31.1
	Dyslipidemia	90,092	31.5	110,562	38.6
	Stroke	12,235	4.3	15,223	5.3
	Ischemic heart disease	36,348	12.7	43,019	15.0
	Cancer	20,510	7.2	25,521	8.9

Note. MVPA: moderate to vigorous physical activity; BMI: body mass index; SBP: systolic blood pressure; DBP: diastolic blood pressure.

**Table 2:** Frequency of moderate to vigorous physical activity at baseline period and subsequent risk of all-cause and CVD mortality.

Variable	Number of Participants	Person-Years	Cases	HR ^a^	95% CI ^a^		*p*-Value
All-cause mortality							
Physical inactivity	137,181	807,247	2728	1.00			
1–2 sessions of MVPA per week	80,598	476,581	733	0.82	0.76	0.89	<0.001
3–4 sessions of MVPA per week	41,178	243,791	522	0.87	0.79	0.96	0.005
≥5 sessions of MVPA per week	27,445	162,719	511	0.80	0.73	0.88	<0.001
CVD mortality							
Physical inactivity	137,181	807,247	536	1.00			
1–2 sessions of MVPA per week	80,598	476,581	133	0.88	0.73	1.07	0.206
3–4 sessions of MVPA per week	41,178	243,791	73	0.70	0.55	0.90	0.005
≥5 sessions of MVPA per week	27,445	162,719	78	0.69	0.54	0.88	0.002

Note. HR, hazard ratio; CI, confidence interval; CVD, cardiovascular disease. ^a^ HR and 95% CI were estimated after adjusting for sex, age, BMI, SBP, DBP, fasting glucose, total cholesterol, alcohol consumption, cigarette smoking status, household income, residential area, disability, and comorbidities.

## Data Availability

Data was obtained from a third party and are not publicly available.

## Supplementary Materials

The following supporting information can be downloaded at: https://www.mdpi.com/article/10.3390/ijerph19010504/s1, Table S1: Changes in frequency of MVPA with subsequent risk of all-cause and CVD mortality after eliminating cases of mortality at first year after follow-up health examination.
