# Supplementary material for: Changes in the Frequency of Moderate-to-Vigorous Physical Activity and Subsequent Risk of All-Cause and Cardiovascular Disease Mortality
Source: Int J Environ Res Public Health. 2022 Jan 3;19(1):504. doi: 10.3390/ijerph19010504 (PMC8744773; doi:10.3390/ijerph19010504)
Supplement: Supplementary file 1 [file ijerph-19-00504-s001.zip › ijerph-1495679-supplementary.pdf]

Supplementary Table S1.Changes in frequency of MVPA with subsequent risk of all-cause and CVD mortality after eliminating cases of mortality at first year after follow-up health examination.

Variables	All-cause mortality		CVD mortality	
	Cases	HR (95% CI)[a]	Cases	HR (95% CI)[a]
Physical inactivity at baseline health examination				
Continual physical inactivity	1,683	1.00	345	1.00
Increase of MVPA from Physical inactivity to 1-2 times per week	299	0.84 (0.74-0.94)	46	0.71 (0.52-0.97)
Increase of MVPA from physical inactivity to 3-4 times per week	154	0.78 (0.66-0.92)	20	0.56 (0.35-0.88)
Increase of MVPA from physical inactivity to ≥5 times per week	163	0.75 (0.63-0.88)	32	0.76 (0.53-1.10)
1-2 times per week of MVPA at baseline health examination				
Decrease of MVPA from 1-2 times per week to physical inactivity	315	1.26 (1.04-1.53)	64	1.43 (0.89-2.31)
Continuously 1-2 times per week of MVPA	179	1.00	27	1.00
Increase of MVPA from 1-2 times per week to 3-4 times per week	77	0.95 (0.73-1.25)	15	1.23 (0.65-2.32)
Increase of MVPA from 1-2 times per week to ≥5 times per week	51	1.02 (0.74-1.40)	4	0.50 (0.17-1.43)
3-4 times per week of MVPA at baseline health examination				
Decrease of MVPA from 3-4 times per week to physical inactivity	193	1.28 (1.00-1.65)	39	3.25 (1.49-7.10)
Decrease of MVPA from 3-4 times per week to 1-2 times per week	78	0.97 (0.73-1.31)	7	1.14 (0.41-3.14)
Continuously 3-4 times per week of MVPA	103	1.00	8	1.00
Increase of MVPA from 3-4 times per week to ≥5 times per week	71	1.15 (0.85-1.56)	8	1.58 (0.59-4.23)
≥5 times per week of MVPA at baseline health examination				
Continuously ≥5 times per week of MVPA	118	1.00	17	1.00
Decrease of MVPA from ≥5 times per week to 3-4 times per week	62	0.99 (0.73-1.35)	6	0.68 (0.27-1.73)
Decrease of MVPA from ≥5 times per week to 1-2 times per week	56	1.08 (0.78-1.49)	6	0.80 (0.31-2.04)
Decrease of MVPA from ≥5 times per week to physical inactivity	207	1.42 (1.12-1.79)	37	1.48 (0.82-2.68)

Abbreviation. CI, confidence interval; CVD, cardiovascular disease; HR, hazard ratio; MVPA, moderate to vigorous physical activity

[a]HR and 95% CI were estimated after adjusting for sex, age, BMI, SBP, DBP, fasting glucose, total cholesterol, alcohol consumption, cigarette smoking status, household income, residential area, disability, and comorbidities
